# Pain relief for femur fractures using Fascia Iliaca Block and IV fentanyl

**DOI:** 10.6026/973206300210567

**Published:** 2025-03-31

**Authors:** Monika Gandhi, Manjeet Singh Meena, Rashpal Singh Gill, Aseem Sharma, Manish Banjare

**Affiliations:** 1 Department of Anesthesiology, M.G.M. Medical College and M.Y. Hospital, Indore, Madhya Pradesh, India

**Keywords:** Fascia iliaca compartment block, intravenous fentanyl, proximal femur fracture, pain management, spinal anesthesia positioning, postoperative analgesia

## Abstract

Proximal femur fractures cause severe pain making spinal anesthesia positioning difficult. Therefore, it is of interest to compare ultrasound-guided Fascia Iliaca Compartment Block (F.I.C.B) with IV fentanyl for pain relief. 100 American Society of Anesthesiologists (ASA) I/II patients were randomly divided into two groups, receiving either Fascia Iliaca Compartment Block (30 ml 0.25% bupivacaine) or IV fentanyl (1 mcg/kg). Visual analog scale (VAS) scores were similar at baseline but significantly lower in Fascia Iliaca Compartment Block group during positioning (2.38 ± 0.49 vs 4.34 ± 0.72, p < 0.0001). Further, Fascia Iliaca Compartment Block also led to faster spinal anesthesia (6.21 ± 0.86 vs 6.96 ± 0.2 min, p < 0.0001) and longer pain relief (4.72 ± 0.78 vs 2.7 ± 0.76 hrs, p < 0.0001) with stable vitals and no significant difference in side effects.

## Background:

Proximal femur fractures, particularly in elderly patients, pose significant challenges in pain management during spinal anesthesia positioning. Uncontrolled pain can hinder optimal positioning, prolong procedures and increase the risk of complications [1, 2]. Traditional methods like intravenous opioids (fentanyl) offer rapid pain relief but are associated with dose-dependent side effects, including respiratory depression, nausea and hemodynamic instability [3]. In contrast, regional techniques such as fascia iliaca compartment block (F.I.C.B) are gaining popularity due to their ability to target the femoral, lateral femoral cutaneous and obturator nerves, offering localized pain control without systemic opioid-related risks [4, 5]. However, comparative data on Fascia Iliaca Compartment Block versus IV fentanyl specifically for positioning pain remain limited [6, 7]. Therefore, it is of interest to evaluate F.I.C.B's role in optimizing spinal anesthesia positioning, improving procedural efficiency and enhancing post-operative pain control [7, 8].

## Materials and Methods:

After obtaining approval from Ethics Committee, 100 patients classified -ASA grade I or II and scheduled for elective surgery were included in the study. All patients underwent preoperative assessments and informed consent was taken before enrollment. Patients were asked to fast for six hours prior to surgery. They were then randomly divided into two groups- Group Fascia Iliaca Compartment Block and Group FENT. In Group F.I.C.B- 30 ml of 0.25% plain bupivacaine was administered using an ultrasound-guided F.I.C.B. In Group FENT-intravenous fentanyl at a dose of 1 mcg/kg body weight was given slowly over two minutes. Both interventions were performed 15 minutes prior to administering the subarachnoid block (SAB). Intravenous access was established for all patients and they were preloaded with Ringer lactate. Baseline vitals, including heart rate, mean arterial pressure, oxygen saturation, respiratory rate and VAS pain scores, were recorded before the interventions. In the Fascia Iliaca Compartment Block group, the block was performed using ultrasound guidance, while in the FENT group, the drug was administered intravenously. Hemodynamic parameters and VAS scores were closely monitored at regular intervals. Once a VAS score of ≤3 was achieved, patients were positioned for the SAB and the ease of positioning was noted. Additional analgesia was given if necessary. A SAB was performed using 0.3 mg/kg of 0.5% bupivacaine. Postoperative vitals were monitored and rescue analgesia (IV paracetamol 15 mg/kg) was given as needed. Adverse effects like nausea, vomiting or hypotension were managed. Study data included VAS scores, positioning ease, SAB time, hemo-dynamics, rescue analgesia timing and adverse events. Analysis used Statistical Package for the Social Sciences (SPSS-25.0), with quantitative data as mean ± SD, categorical data as percentages and significance at p < 0.05.

## Results:

The demographic and clinical parameters were well-balanced between groups as in Table 1. The mean age was 47.88 ± 13.04 years in the Fascia Iliaca Compartment Block group and 45 ± 13.4 years in the FENT group (p = 0.279). Gender distribution was similar, with 72% males in Fascia Iliaca Compartment Block and 68% in FENT (p = 0.663). ASA Grade I-II proportions were comparable (p = 0.689) and there was no significant weight difference (p = 0.931). Table 2 Fascia Iliaca Compartment Block provided superior pain relief at critical time points. Just 2 minutes post-intervention, VAS scores were higher in the Fascia Iliaca Compartment Block group (6.72 ± 0.99) than FENT (5.9 ± 0.91, p < 0.0001), but by 15 minutes, Fascia Iliaca Compartment Block had significantly lower scores (2.54 ± 0.5 vs. 3 ± 0, p < 0.0001). During positioning the difference was even more pronounced VAS scores in the Fascia Iliaca Compartment Block group were 2.38 ± 0.49 compared to 4.34 ± 0.72 in the FENT group (p < 0.0001) highlighting its efficacy in spinal anesthesia positioning. Fascia Iliaca Compartment Block improved spinal anesthesia efficiency the time required to complete SAB was 6.21 ± 0.86 min vs. 6.96 ± 0.2 min in FENT (p < 0.0001). More importantly, Fascia Iliaca Compartment Block patients experienced significantly prolonged analgesia, requiring their first rescue dose at 4.72 ± 0.78 hrs, compared to 2.7 ± 0.76 hrs in FENT (p < 0.0001). These findings from Table 3 underscore F.I.C.B's ability to enhance procedural efficiency while extending pain relief well beyond fentanyl's duration. Vitals remained clinically stable in both groups throughout the study as in Table 4. At baseline, heart rate (HR), mean arterial pressure (MAP), oxygen saturation (SpO_2_) and respiratory rate (RR) were comparable. No statistically significant differences were observed at 0 min, 5 min, or 15 min post-intervention. This confirms that Fascia Iliaca Compartment Block does not introduce additional hemodynamic variability compared to fentanyl. Adverse effects in Table 5 were slightly higher in the FENT group compared to the Fascia Iliaca Compartment Block group, but the differences were not statistically significant. Nausea was reported in 5 patients (10%) in the FENT group versus 2 patients (4%) in the Fascia Iliaca Compartment Block group (p = 0.436). Vomiting occurred in 2 patients (4%) in the FENT group and none in the Fascia Iliaca Compartment Block group (p = 0.495). Hypotension was observed in 3 patients (6%) in the FENT group and in 1 patient (2%) in the Fascia Iliaca Compartment Block group (p = 0.617).

## Discussion:

This study compared ultrasound-guided Fascia Iliaca Compartment Block and I.V fentanyl for pain relief during positioning in femur fracture patients. Table 1 confirmed both groups were comparable in age, gender, ASA grade and weight, minimizing baseline differences that could affect outcomes. Fascia Iliaca Compartment Block showed better pain control with a lower VAS scores than FENT during positioning 2.38 ± 0.49 vs 4.34 ± 0.72 p < 0.0001 though baseline scores were similar (Table 2). This helped in making positioning easier for spinal anesthesia. Pain relief pattern varied in both groups. At 2 and 5 minutes post-intervention, FENT had lower pain scores (5.9 ± 0.91 and 3.8 ± 0.73) compared to Fascia Iliaca Compartment Block (6.72 ± 0.99 and 4.84 ± 0.62, p < 0.0001). But by 15 minutes, Fascia Iliaca Compartment Block showed better pain relief. This pattern suggests fentanyl acts fast initially while Fascia Iliaca Compartment Block provides prolonged analgesia [10-11]. Findings are similar to Madabushi *et al.* who found Fascia Iliaca Compartment Block improved pain relief and positioning for spinal anesthesia better than I.V fentanyl in femur fracture surgeries [10]. But not all studies agree. Some studies found no major difference between Fascia Iliaca Compartment Block and femoral nerve block for positioning [9-11]. These variations could be due to lower bupivacaine concentration (0.3%) and shorter waiting time before positioning (15 minutes). Table 3 shows the time required for spinal anesthesia was significantly shorter in the Fascia Iliaca Compartment Block group (6.21 ± 0.86 min) compared to FENT (6.96 ± 0.2 min, p < 0.0001). Better pain relief likely helped smoother positioning leading to faster spinal anesthesia [12-13]. In busy hospitals this can save time and help complete more procedures efficiently [14]. Another benefit of Fascia Iliaca Compartment Block seen in Table 3 was extended postoperative pain relief. Patients in the Fascia Iliaca Compartment Block group needed rescue analgesia much later (4.72 ± 0.78 hrs) compared to FENT (2.7 ± 0.76 hrs, p < 0.0001). This longer relief may reduce early opioid use and its side effects [4, 5 and 16]. These findings align with Guo *et al.* whose meta-analysis showed Fascia Iliaca Compartment Block reduced opioid intake and pain scores [15]. Yang *et al.* also found Fascia Iliaca Compartment Block lowered pain at different time points and reduced morphine consumption at 24 hours [16]. However, Kristin *et al.* found Fascia Iliaca Compartment Block improved self-reported pain but didn't significantly lower opioid use [17]. This variation shows pain management is complex and needs more study [18]. Both groups showed stable hemodynamics throughout as seen in Table 4. Heart rate, blood pressure, oxygen saturation and respiratory rate showed no significant difference at baseline, 0 minutes and 15 minutes post-intervention (all p-values > 0.05) [19, 20]. These findings indicate Fascia Iliaca Compartment Block is safe for patients including elderly individuals with other illnesses [20]. Fascia Iliaca Compartment Block also showed a trend toward fewer adverse effects but differences were not statistically significant. Table 5 shows nausea occurred in 4% of Fascia Iliaca Compartment Block patients vs 10% in FENT (p = 0.436), vomiting in 0% vs 4% (p = 0.495) and hypotension in 2% vs 6% (p = 0.617). Though these findings are promising, larger studies are needed to confirm Fascia Iliaca Compartment Block's safety [17]. Other studies like Aprato *et al.* suggest intra-articular hip injections might be better in some cases [21]. Sample size was sufficient for primary outcomes but may not have detected smaller differences in secondary outcomes like side effects. Long term recovery and mobility were not studied [17]. Also different regional blocks may be useful in some cases. Future research should compare Fascia Iliaca Compartment Block with other regional techniques and include a placebo group [18]. Combining Fascia Iliaca Compartment Block with systemic analgesia might offer even better results [21]. Larger trials with long-term follow-up are needed to confirm Fascia Iliaca Compartment Block's role in pain management and its long-term effects.

## Conclusion:

Fascia Iliaca Compartment Block significantly improved pain relief and spinal positioning efficiency in femur fracture patients compared to IV fentanyl. It also extended postoperative analgesia reducing early opioid use without compromising hemodynamic stability. These findings reinforce the role of Fascia Iliaca Compartment Block as a practical, evidence based advancement in regional anesthesia for fracture pain management.

## Figures and Tables

**Table 1:** Baseline demographic and clinical characteristics

**Characteristic**	**Group Fascia Iliaca Compartment Block (n = 50)**	**Group FENT (n = 50)**
**Age (years)**		
- 48	5 (10%)	9 (18%)
- 71	12 (24%)	12 (24%)
- 91	10 (20%)	12 (24%)
- 111	12 (24%)	9 (18%)
- 126	11 (22%)	8 (16%)
- Mean ± SD	47.88 ± 13.04	45 ± 13.4
**Gender**		
- Female	14 (28%)	16 (32%)
- Male	36 (72%)	34 (68%)
**ASA Grade**		
- Grade I	25 (50%)	23 (46%)
- Grade II	25 (50%)	27 (54%)
Weight (kg)	60.22 ± 9.59	60.42 ± 13.07

**Table 2:** VAS scores after intervention and during positioning

**Time Point**	**Group Fascia Iliaca Compartment Block (Mean ± SD)**	**Group FENT (Mean ± SD)**	**p-value**
**Just After Intervention**			
- At 2 minutes	6.72 ± 0.99	5.9 ± 0.91	<0.0001
- At 5 minutes	4.84 ± 0.62	3.8 ± 0.73	<0.0001
- At 10 minutes	3.08 ± 0.4	3.04 ± 0.2	0.525
- At 15 minutes	2.54 ± 0.5	3 ± 0	<0.0001
**During Positioning**			
- Baseline	8.58 ± 0.88	8.54 ± 0.65	0.797
- 15 minutes	2.54 ± 0.5	3 ± 0	<0.0001
- During positioning	2.38 ± 0.49	4.34 ± 0.72	<0.0001

**Table 3:** Procedural and pain management outcomes

**Outcome**	**Group Fascia Iliaca Compartment Block (n = 50)**	**Group FENT (n = 50)**	**p-value**
Time to Perform SAB (mins)	6.21 ± 0.86	6.96 ± 0.2	<0.0001
Time to Rescue Analgesia (hrs)	4.72 ± 0.78	2.7 ± 0.76	<0.0001

**Table 4:** Changes in vitals after intervention

**Time Point**	**Group**	**HR (bpm)**	**p-value**	**MAP (mmHg)**	**p-value**	**SpO_2_ (%)**	**p-value**	**RR (bpm)**	**p-value**
Baseline	F.I.C.B	82.76 ± 10.81	0.165	96.68 ± 6.38	0.163	99.2 ± 1.07	0.681	15.78 ± 1.73	0.169
	FENT	80.48 ± 3.86		95.04 ± 5.22		99.28 ± 0.86		16.18 ± 1.08	
0 minutes	F.I.C.B	84.98 ± 13.04	0.116	96.68 ± 9.61	0.737	99.74 ± 0.66	0.104	15.94 ± 2.06	0.613
	FENT	81.88 ± 4.33		96.12 ± 6.81		99.92 ± 0.4		16.1 ± 0.84	
At 15 minutes	F.I.C.B	80.88 ± 9.13	0.339	93.24 ± 9.94	0.12	99.66 ± 0.87	0.278	15.32 ± 2.15	0.092
	FENT	82.26 ± 4.42		95.88 ± 6.54		99.82 ± 0.56		15.92 ± 1.24	

**Table 5:** Adverse effects in both groups

**Adverse Effect**	**Group Fascia Iliaca Compartment Block (n = 50)**	**Group FENT (n = 50)**	**p-value**
Nausea	2 (4%)	5 (10%)	0.436
Vomiting	0 (0%)	2 (4%)	0.495
Hypotension	1 (2%)	3 (6%)	0.617
